# Supplementary material for: In situ edge engineering in two-dimensional transition metal dichalcogenides
Source: Nat Commun. 2018 May 24;9:2051. doi: 10.1038/s41467-018-04435-x (PMC5967346; doi:10.1038/s41467-018-04435-x)
Supplement: Supplementary file 1 — Supplementary Information [file 41467_2018_4435_MOESM1_ESM.pdf]

Supplementary Information for

In situ Edge Engineering in Two-dimensional Transition Metal Dichalcogenides

Sang et al.

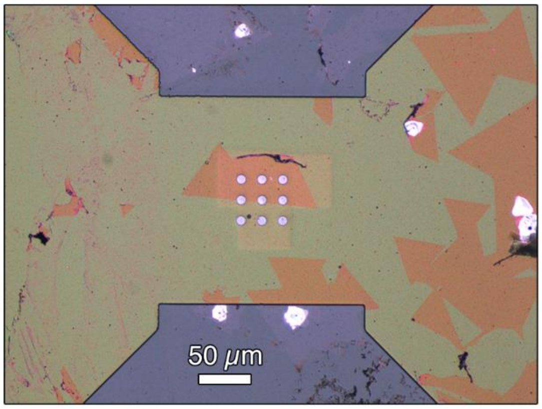

Supplementary Figure 1 | A light optical image of a Protochips heating e-chip partially covered by $Mo_{0.95}W_{0.05}Se_2$ monolayer flakes. The square area in the center containing the nine electron-transparent holes is the region for local resistive heating and *in situ* observations of edge reconstructions.

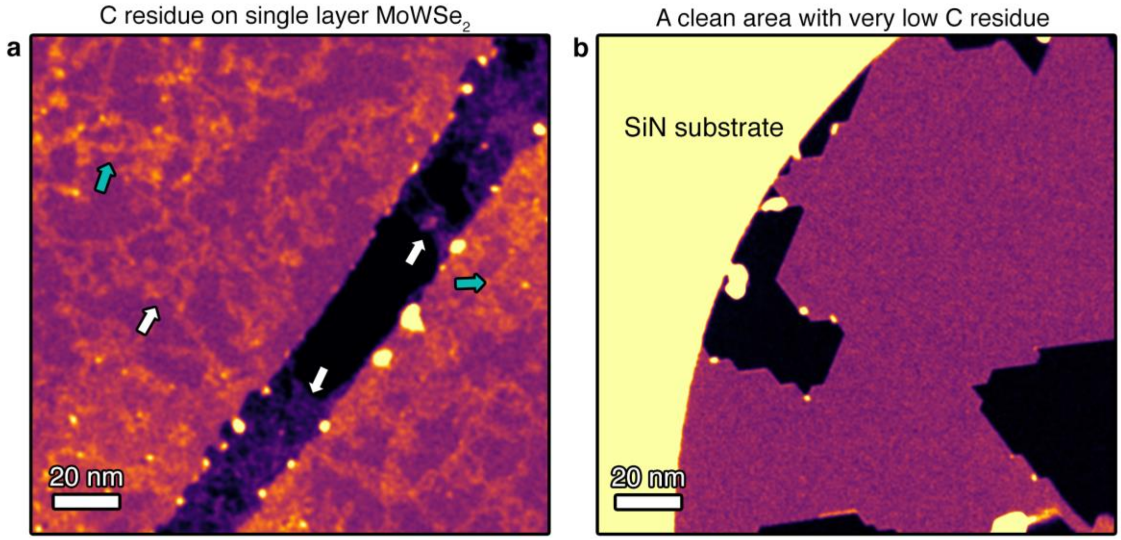

Supplementary Figure 2 | Concentration variation of carbon residue on monolayer $Mo_{0.95}W_{0.05}Se_2$ flakes. **a**, Low magnification HAADF-STEM image of monolayer $Mo_{0.95}W_{0.05}Se_2$ from an area with random distribution of C residue. The orange network of carbon residue is observed. Several areas with moderate carbon residue are indicated by white arrows. Two areas with brighter C residue contrast are indicated by cyan arrows. **b**, Low magnification HAADF-STEM image of monolayer $Mo_{0.95}W_{0.05}Se_2$ from an area without noticeable C residue, as evidenced by the very uniform purple contrast of the flake. The uniform yellow contrast on the left is from SiN substrate.

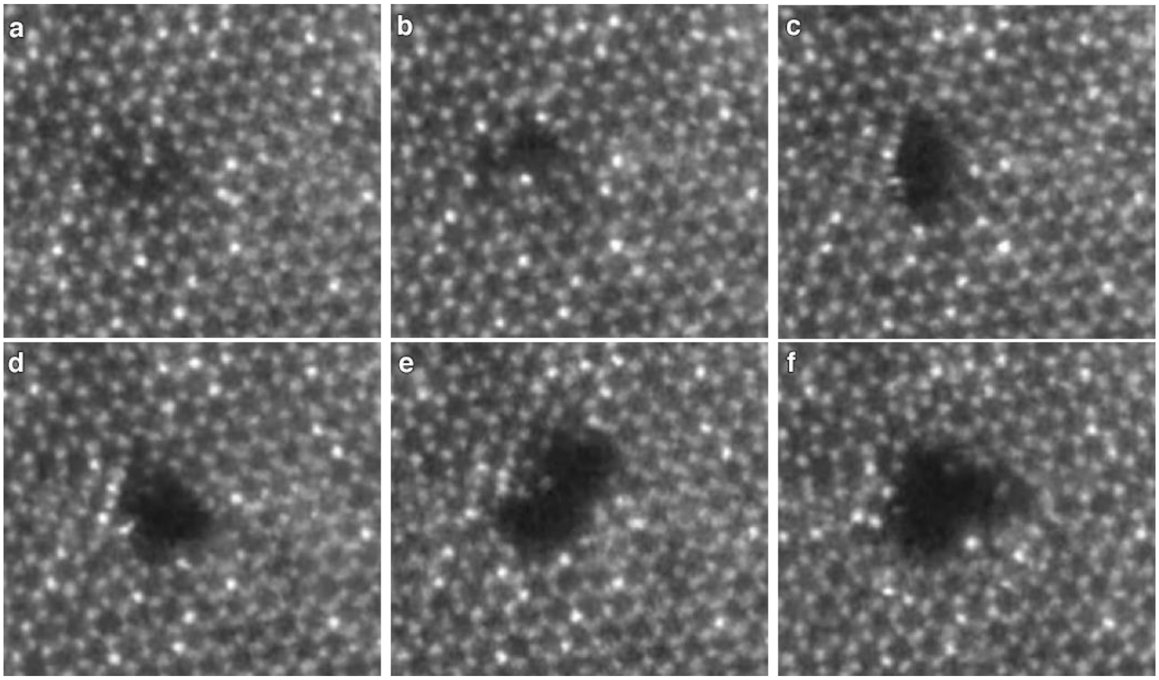

Supplementary Figure 3 | HAADF-STEM images showing the initial stages of the etching process. A pore forms from an area with Se vacancies. From **a**–**f**, the time interval between the acquisition of each image is 9 s.

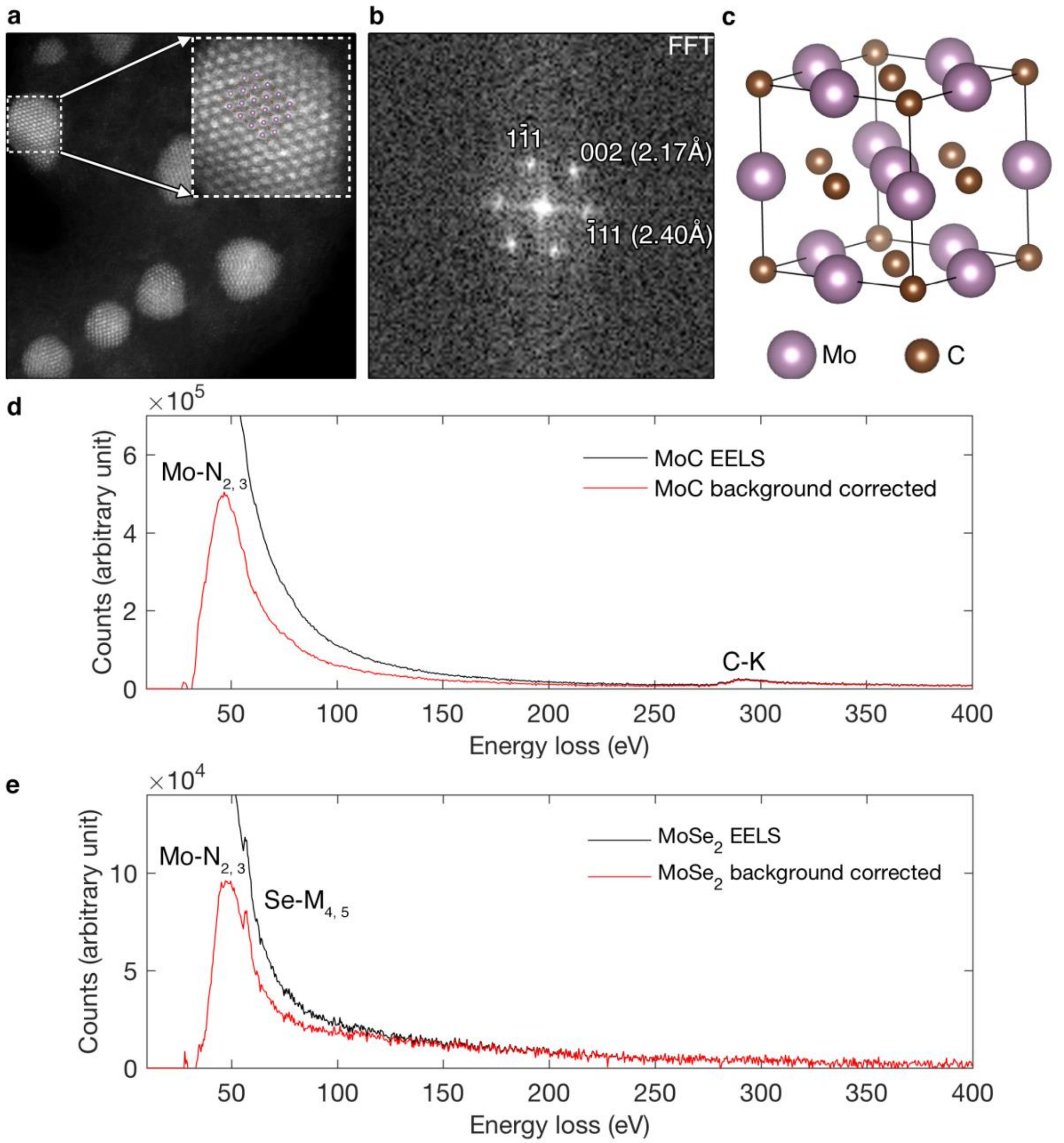

Supplementary Figure 4 | Formation of MoC nanoparticles during *in situ* heating. a, HAADF-STEM image of the nanoparticles. The lattice is in agreement with the NaCl-type MoC structure. Inset is the enlarged view with an overlaid atomic model of MoC. **b**, FFT image corresponding to the inset in **a**. The lattice constant matches the NaCl-type MoC. **c**, Atomic model of MoC. **d-e**, Electron energy loss spectroscopy (EELS) acquired from a MoC nanoparticle (**d**) and clean $MoSe_2$ single layer flake (**e**). The elemental edges Mo-$N_{2,3}$ at 35 eV, Se-$M_{4,5}$ at 57 eV, and C-K at 284 eV are labeled. The EEL spectrum from MoC confirms absence of Se in MoC particles.

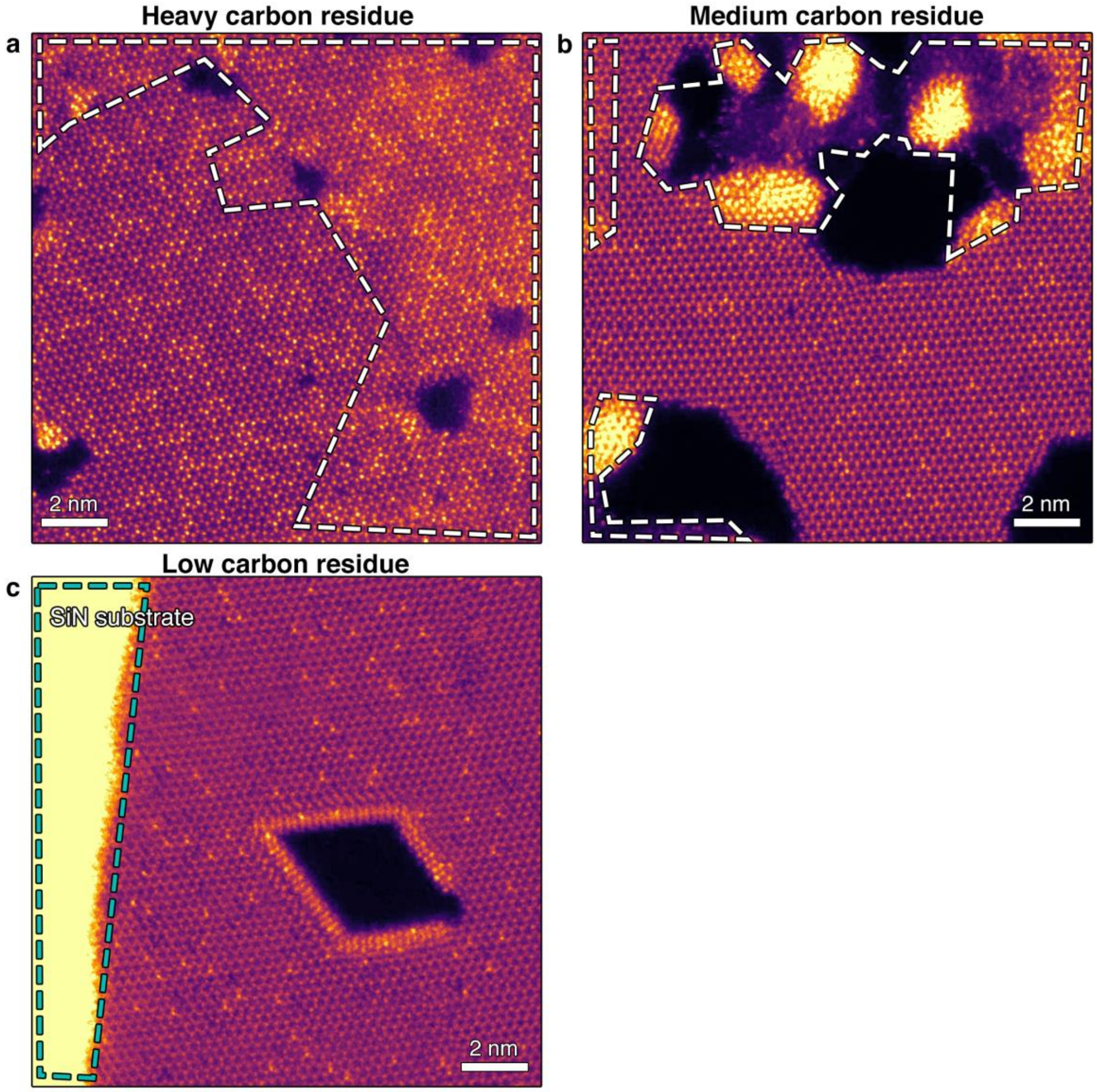

Supplementary Figure 5 | STEM images with different C residue concentration. a-c, The full, uncropped frames of initial STEM images in Fig. 1**d-f**, respectively. Areas with carbon residue are identified by the higher brightness and are enclosed by white dashed lines. The heavy C residue area (**a**) has the largest area of carbon residue with the right half area completedly covered by the C residue. The medium C residue area (**b**) has contamination mainly at the top. The low C residue area (**c**) shows uniform contrast without noticeable C residue.

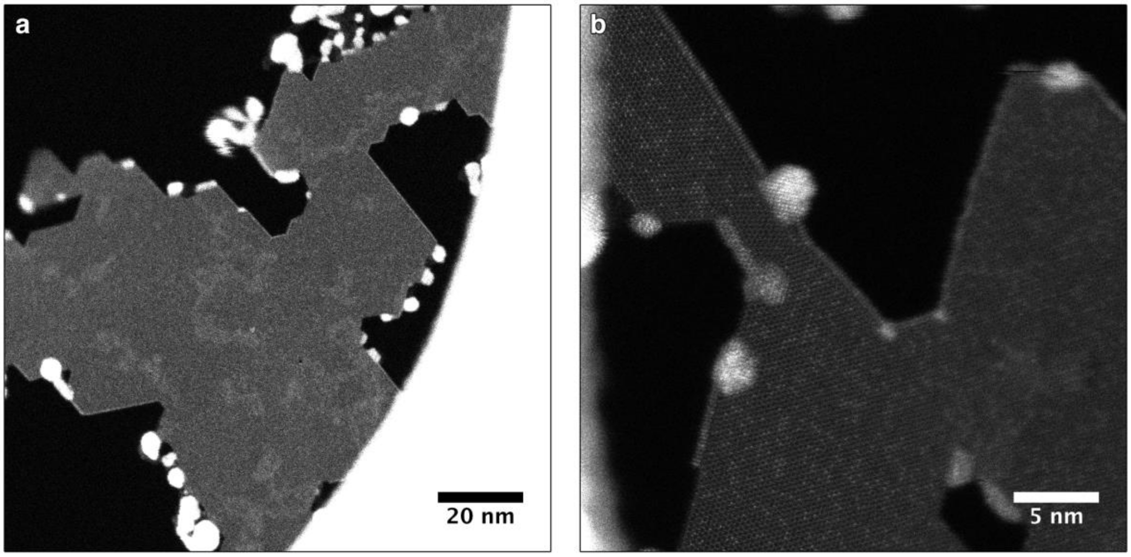

Supplementary Figure 6 | Formation of pores and NW-terminated edges without beam irradiation. a-b, Low magnification HAADF-STEM images of the monolayer $Mo_{0.95}W_{0.05}Se_2$ after heating at 500 °C without e-beam irradiation. STEM images were acquired from areas that were far away from the e-beam during 2h heating. The monolayer shows similar etched and faceted pores that are mostly covered by NW-terminated edges, indicating that the etching and edge reconstruction can happen solely through thermal exposure.

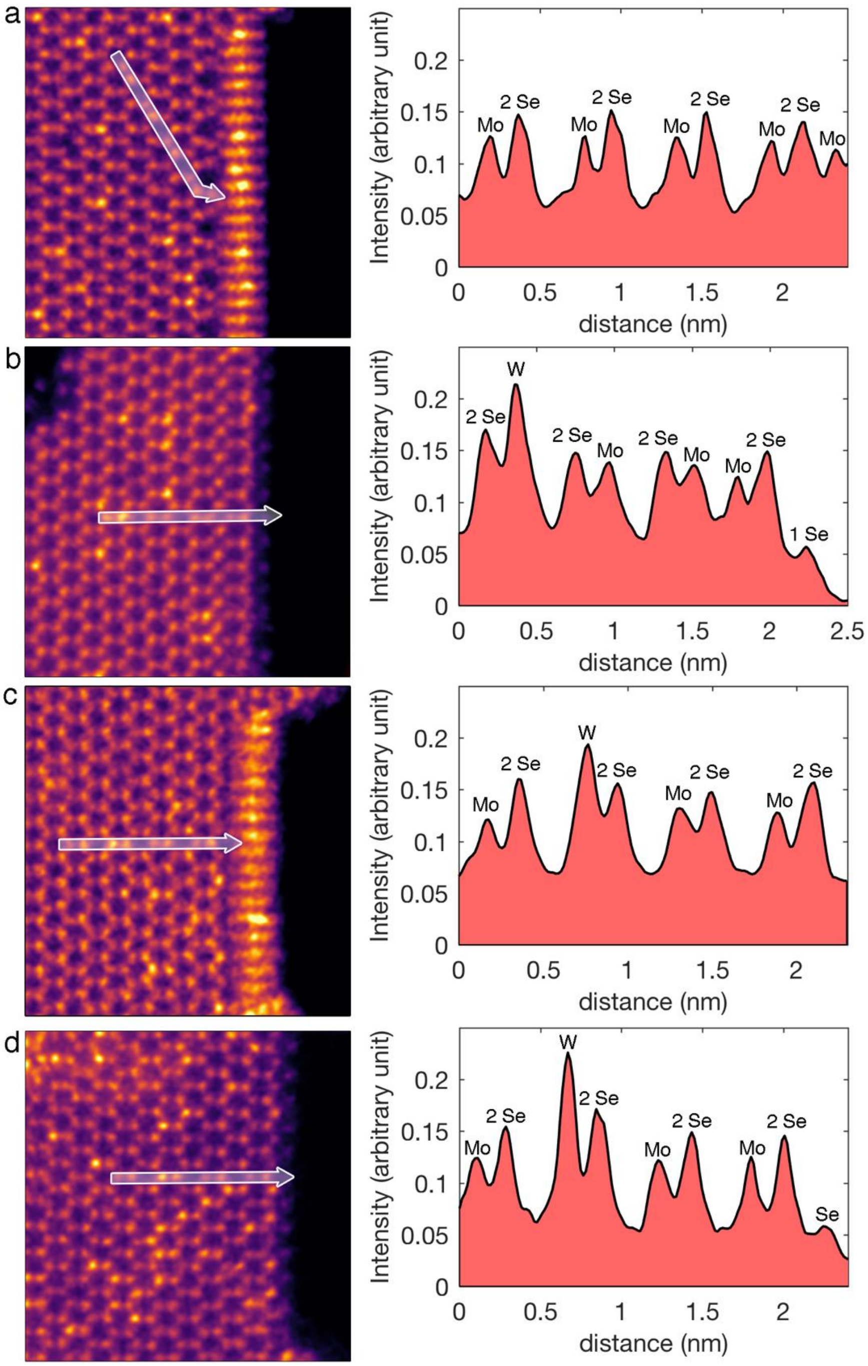

Supplementary Figure 7 | Atomic column identification from intensity line profiles of different edges structures. a-d, Intensity line profiles of the edge structures ZZSe-Mo-NW30, ZZSe-GB4-Se, ZZMo-NW30, and ZZMo-Se as shown in Fig. 2a-d. The intensity line profiles are plotted along the white arrow from the bulk to the edge.

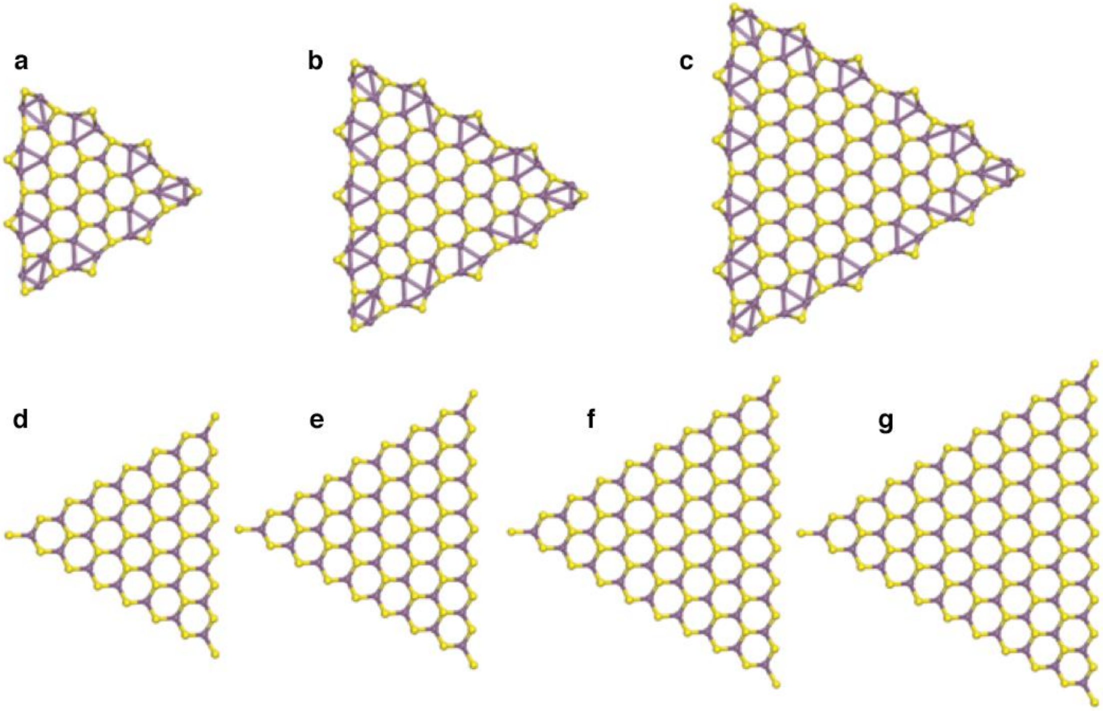

Supplementary Figure 8 | Reference structure for edge formation energy calculation using DFT. Triangular domains used to calculate formation energy of reference edges for Se-oriented ZZSe edges (**a**-**c**) and Mo-oriented ZZMo edges (**d**-**g**) orientation in DFT calculations**.**

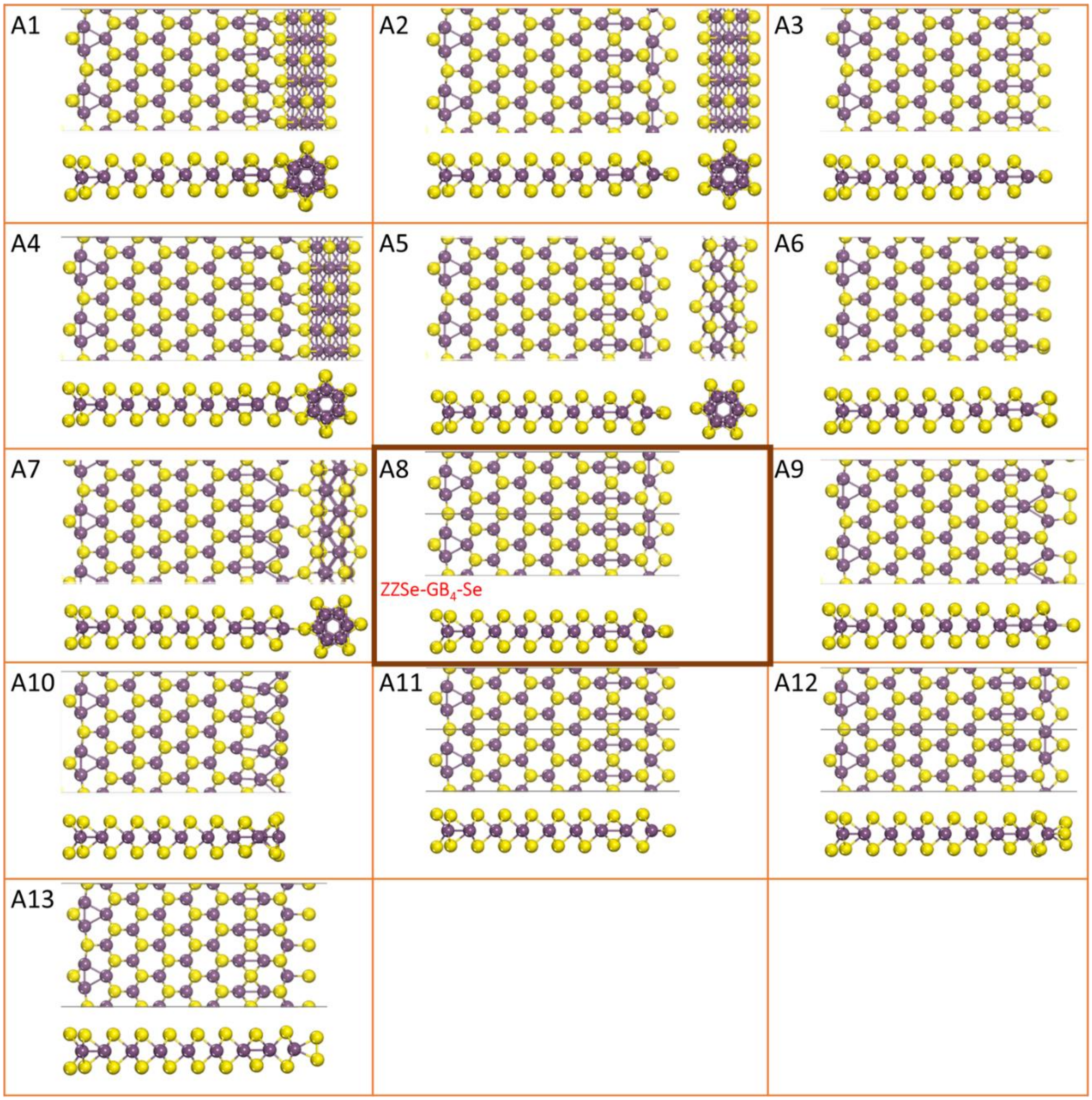

Supplementary Figure 9 | Optimized structures of ZZSe edges (right edge) with 4-fold rings as domain boundaries near the edges (GB4), denoted as A1 to A13. The detailed configurations are: A1: ZZSe-GB4-NW0, A2: ZZSe-GB4-SeMo-Se-NW30, A3: ZZSe-GB4-Se, A4: ZZSe-GB4-SeMo-NW30, A5: ZZSe-GB4-SeMo-Se-NW0, A6: ZZSe-GB4-Se2, A7: ZZSe-GB4-SeMo-NW0, A8: ZZSe-GB4-SeMo-Se, A9: ZZSe-GB4-SeMo-Se57, A10: ZZSe-GB4-SeM0, A11: ZZSe-GB4-SeMo-Se-unreconstructed, A12: ZZSe-GB4-SeMo-1.5Se, A13: ZZSe-GB4-SeMo-Se2. The stable structure A8 is outlined by bold black box.

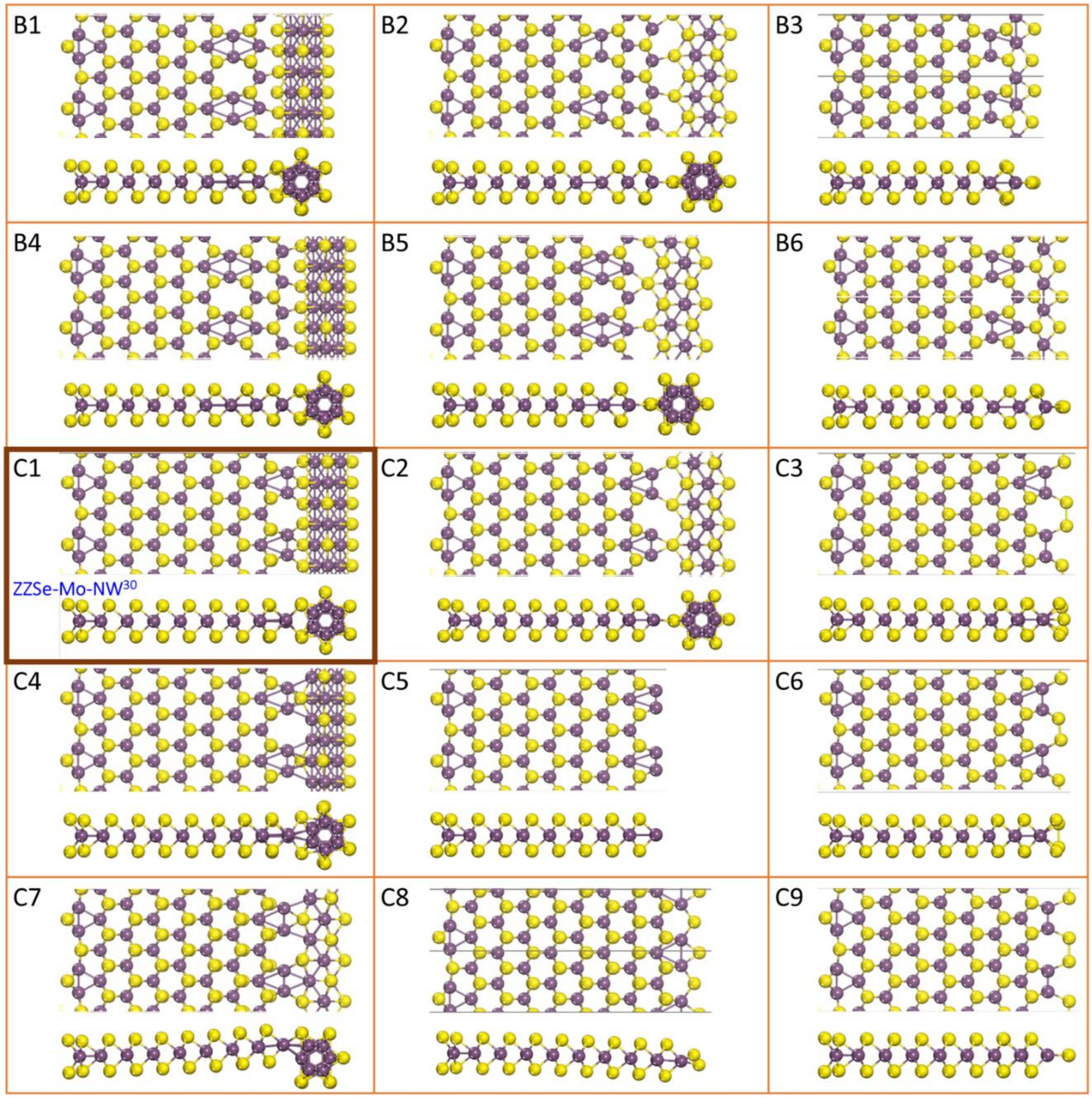

Supplementary Figure 10 | Optimized structures of ZZSe edges (right edge), with 5/8-fold rings as domain boundaries near the edges (GB585), denoted as B1 to B6, and with Mo-dimers near the edges, denoted as C1 to C9. The detailed configurations are: B1: ZZSe-GB585-NW30, B2: ZZSe-GB585-SeMo-NW0, B3: ZZSe-GB585-Se, B4: ZZSe-GB585-SeMo-NW30, B5: ZZSe-GB585-NW0, B6: ZZSe-GB585-SeMo-Se, C1: ZZSe-Mo-NW30, C2: ZZSe-Mo-NW0, C3: ZZSe-Mo-Se3, C4: ZZSe-Mo-NW30-delete4Se, C5: ZZSe-Mo, C6: ZZSe-Mo-Se4, C7: ZZSe-Mo-NW0-delete3Se, C8: ZZSe-Mo-Se, C9: ZZSe-Mo-Se2. The stable structure C1 is outlined by bold black box.

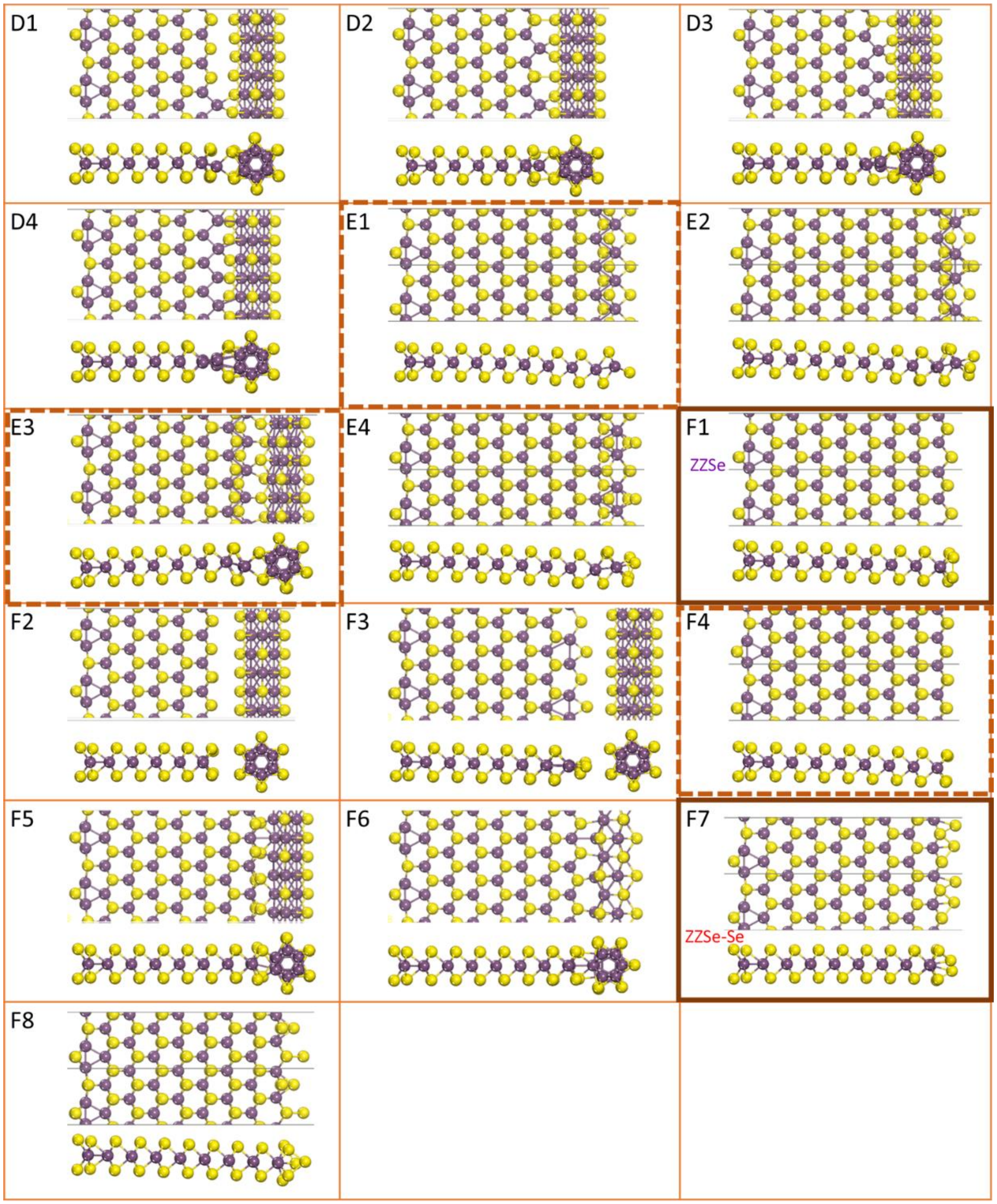

Supplementary Figure 11 | Optimized structures of ZZSe edges (right edge), with different Se2 pair substituted by Mo atoms near the NW, denoted as D1 to D4, with zigzag Mo chains (1T' phase) near the edges, denoted as E1 to E4, and the rest denoted as F1 to F8. The names of them are: D1: ZZSe-Se-to-1Mo-NW0, D2: ZZSe-Se-to-2Mo-NW0, D3: ZZSe-Se-to-3Mo-NW0, D4: ZZSe-Se-to-4Mo-NW0, E1: ZZSe-1T-Se, E2: ZZSe-1T-1.5Se, E3: ZZSe-1T-NW10, E4: ZZSe-1T-2Se, F1: ZZSe, F2: ZZSe-NW30, F3: ZZSe-del4Se-NW30, F4: ZZSe-no-reconstruction, F5: ZZSe-NW30-del6Se, F6: ZZSe-NW0-del3Se, F7: ZZSe-Se, F8: ZZSe-Se-middle. The stable edge structures F1 and F7 are outlined by bold black boxes.

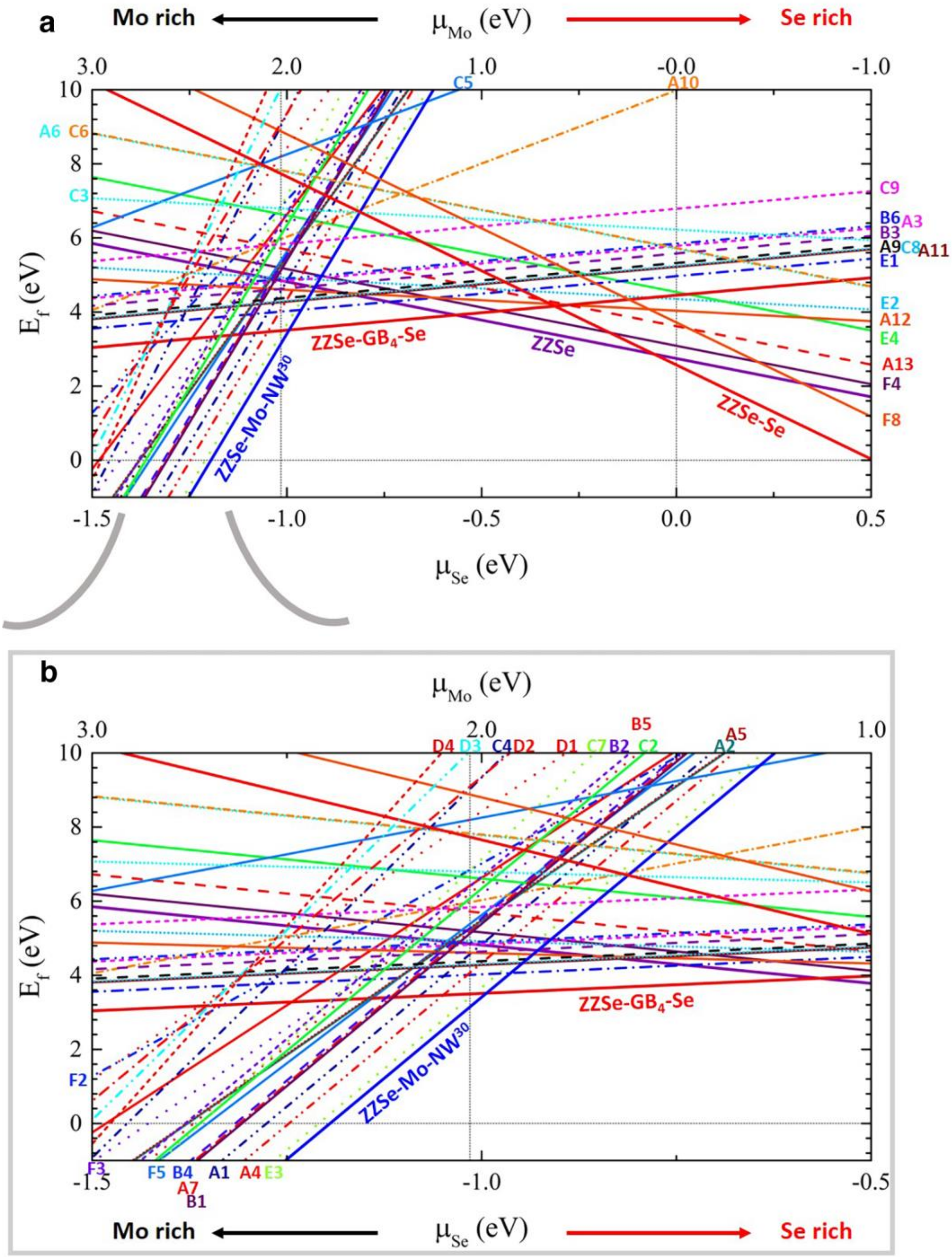

Supplementary Figure 12 | Dependence of formation energy of ZZSe edges on Mo and Se chemical potentials. a, Formation energy of ZZSe edges as a function of Mo and Se chemical potentials. **b**, An enlarged view of **a**.

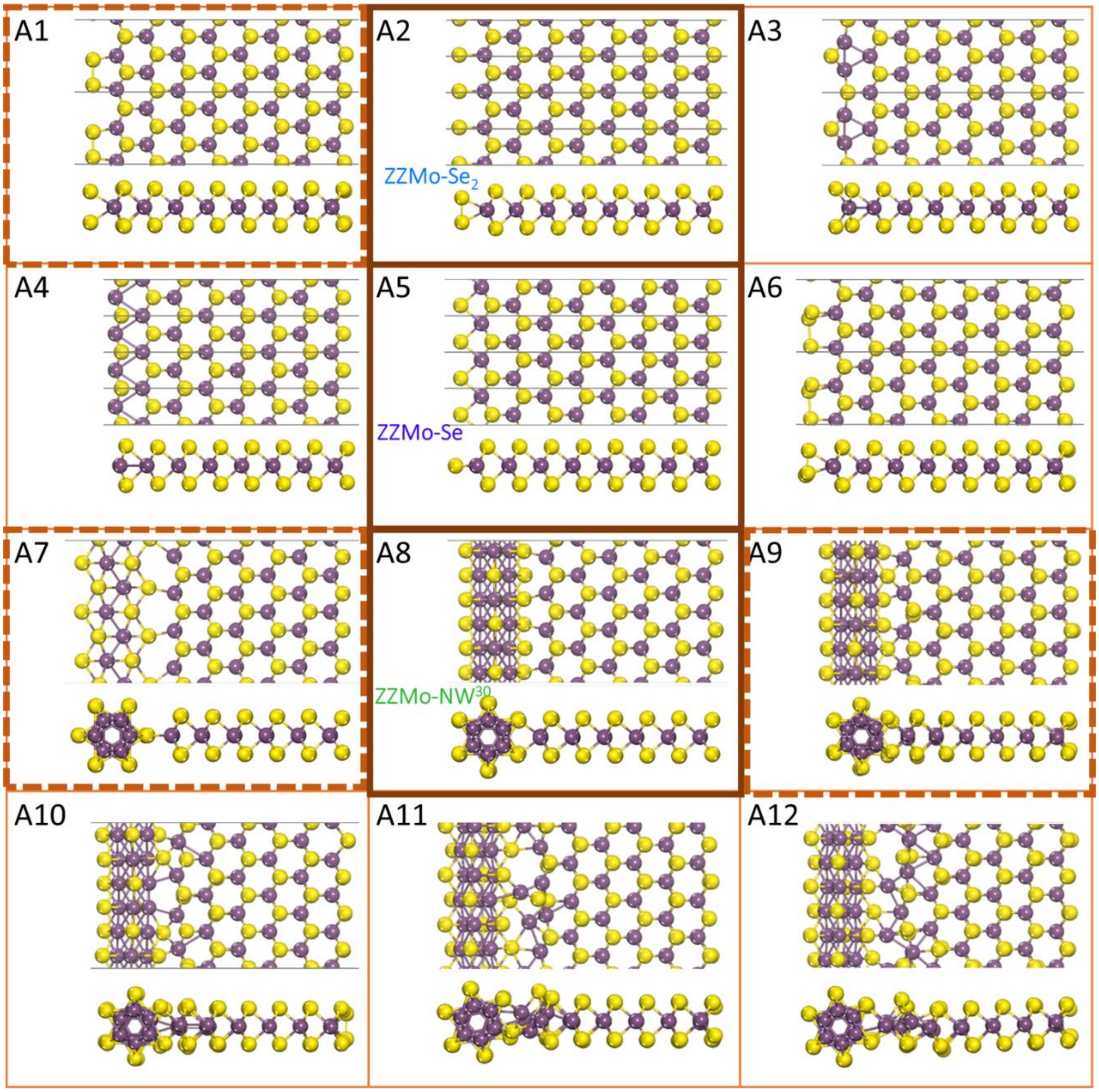

Supplementary Figure 13 | Optimized structures of ZZMo edges (left edge), denoted as A1 to A12. The names of them are: A1: ZZMo-2Se-horizontal-dimer, A2: ZZMo-2Se-vertical-dimer, A3: ZZMo-reconstruction, A4: ZZMo-slight-reconstruction, A5: ZZMo-Se, A6: ZZMo-1.5Se, A7: ZZMo-NW0, A8: ZZMo-NW30, A9: ZZMo-del2Se-NW0, A10: ZZMo-del2Se-NW0-del2Se, A11: ZZMo-add3Mo-NW0, A12: ZZMo-add3Mo-NW30. The stable edge structures A2, A5, and A8 are outlined by bold black boxes.

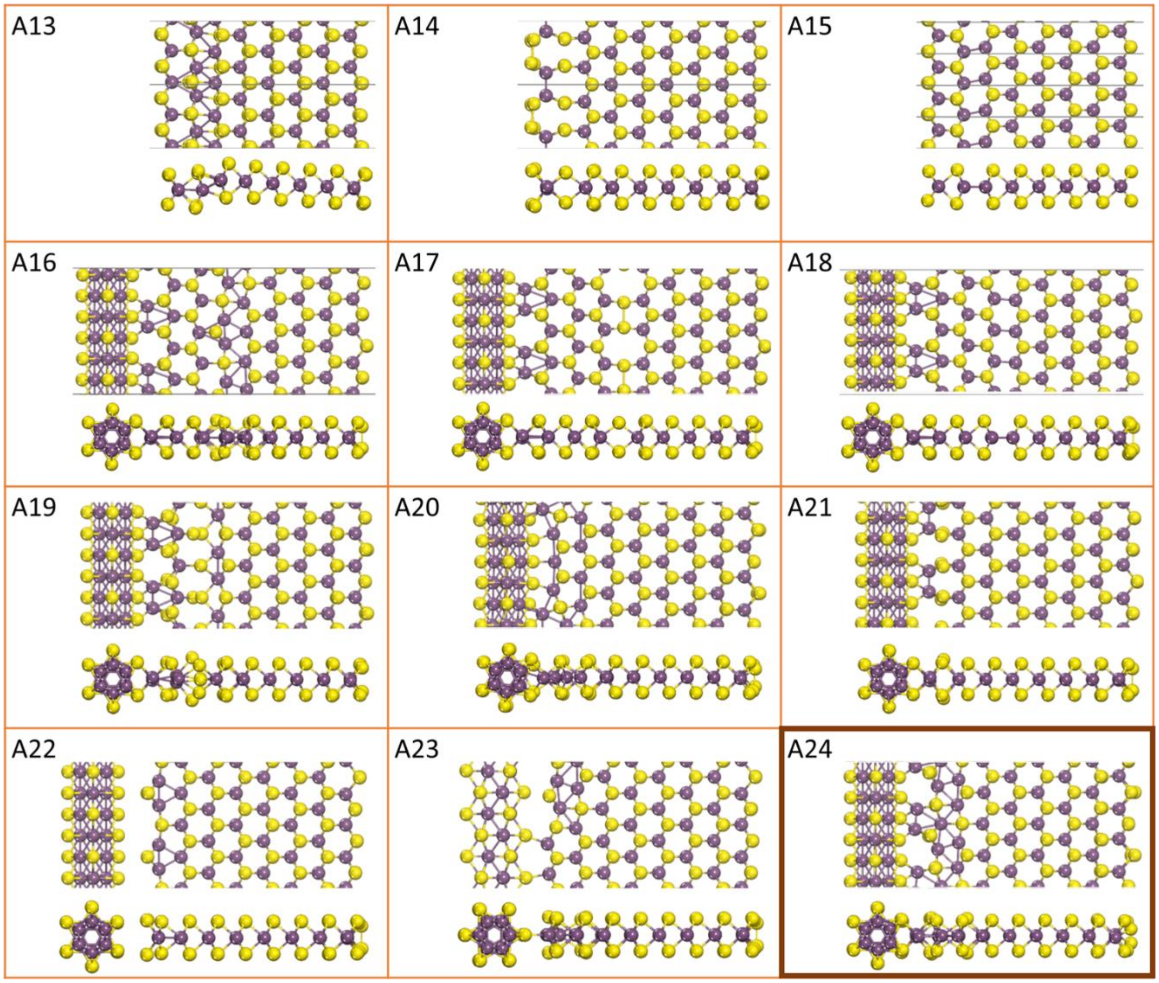

Supplementary Figure 14 | Optimized structures of ZZMo edges (left edge), denoted as A13 to A24. The names of them are: A13: ZZMo-GB-Mo-chain-Se, A14: ZZMo-GB-568, A15: ZZMo-GB-Mo-dimer-Se, A16: ZZMo-GB-Mo-chain-NW30, A17: ZZMo-GB-sDimer-558, A18: ZZMo-GB-Mo-dimer-NW30, A19: ZZMo-GB4-Mo-NW30, A20: ZZMo-reconstruction-57-mmssm-NW0, A21: ZZMo-reconstruction-57-msms-NW0, A22: ZZMo-reconstruction-Se-NW0, A23: ZZMo-reconstruction-Se-NW30, A24: ZZMo-reconstruction-57-mmssm-add2Mo-NW0. The stable edge structure A24 is outlined by a bold black box.

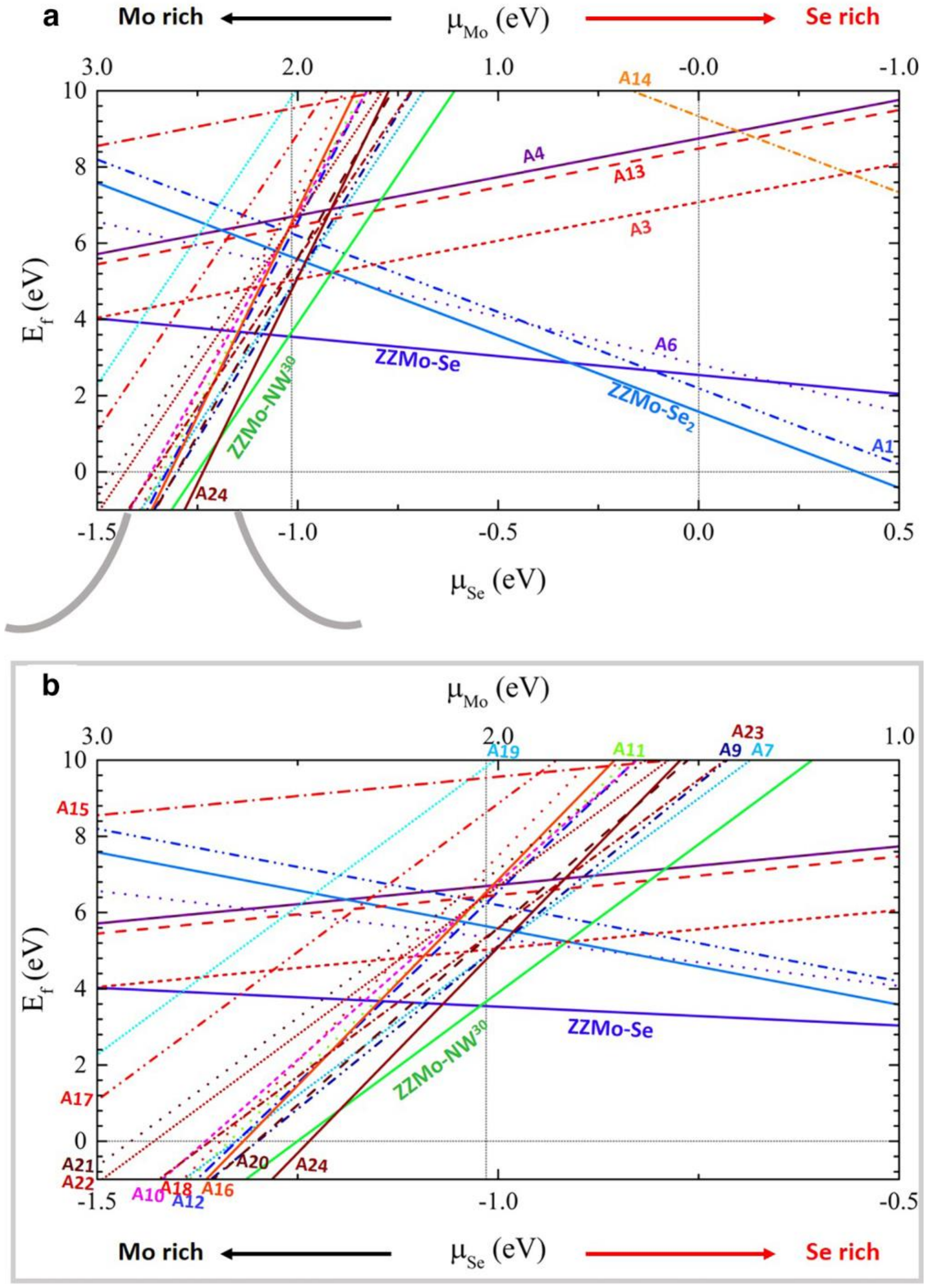

Supplementary Figure 15 | Dependence of formation energy of ZZMo edges on Mo and Se chemical potentials. **a**, Formation energy of ZZMo edges as a function of Mo and Se chemical potentials. **b**, An enlarged view of (**a**).

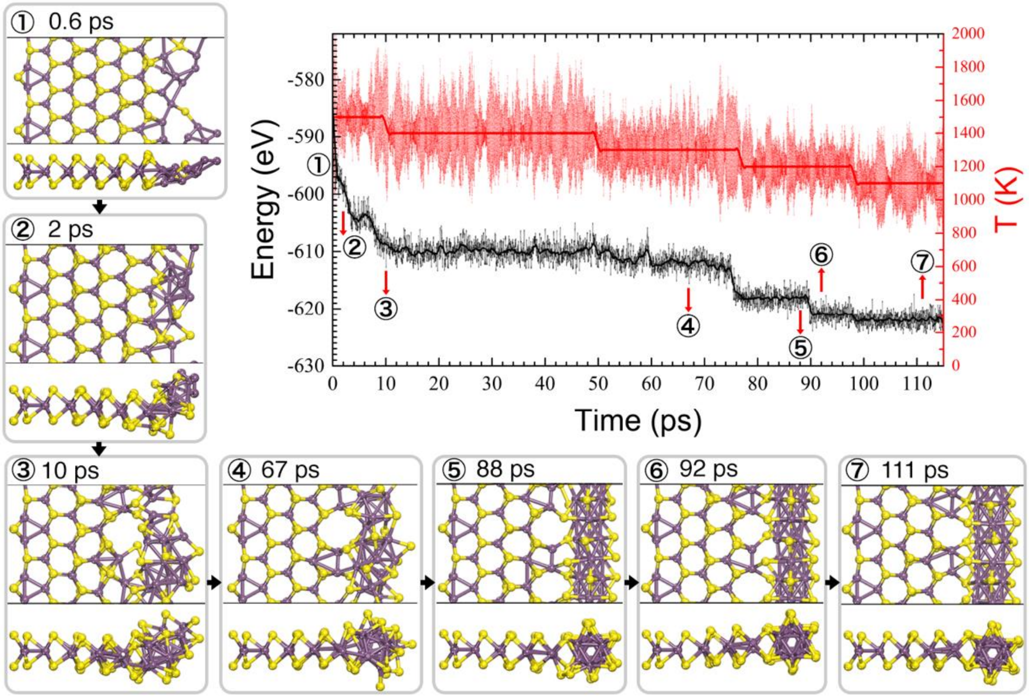

Supplementary Figure 16 | AIMD simulation of the edge energy and temperature as a functional of time in the process of NW evolution. The temperature data (red) and energy data (black) are plotted for every time step ($\Delta t = 1$ fs), and the corresponding thicker lines are smoothed data to guide the eye. The energy of the system keeps decreasing, indicating increased stability. Top (up) and front (down) views of snapshots at different steps are shown under the plot. Compared with Figure 3 in the main manuscript, more snapshots are displayed here.

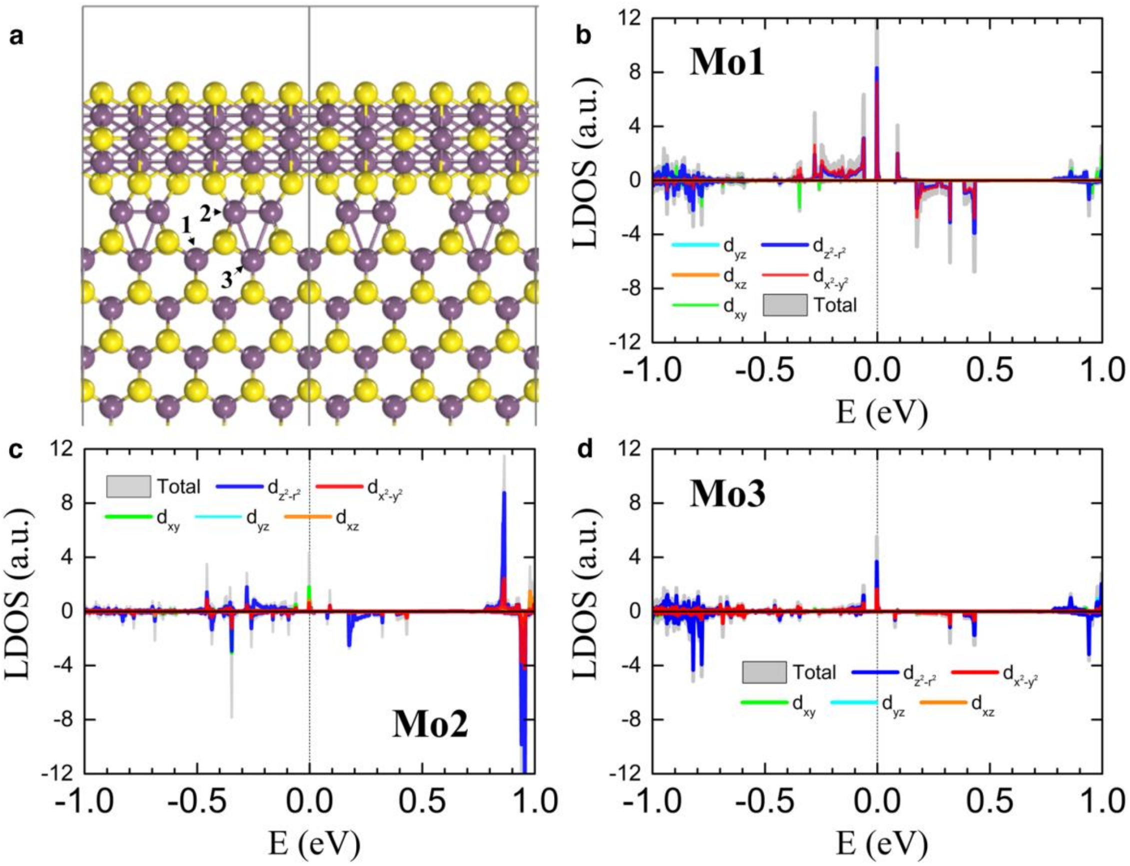

Supplementary Figure 17 | Magnetic properties of the ZZSe-Mo-NW30 edge. a, the three symmetrically inequivalent Mo atoms in a supercell that are spin-polarized, denoted as Mo1, Mo2 and Mo3, which contribute to magnetization by 0.590 μ_B, 0.101 μ_B, and 0.053 μ_B, per atom, respectively. **b**, The LDOS projected on Mo1. **c**, The LDOS projected on Mo2. **d**, The LDOS projected on Mo3. The spin-polarization is mainly contributed by the d_{z2}, d_{x2-y2} and d_{xy} orbitals.

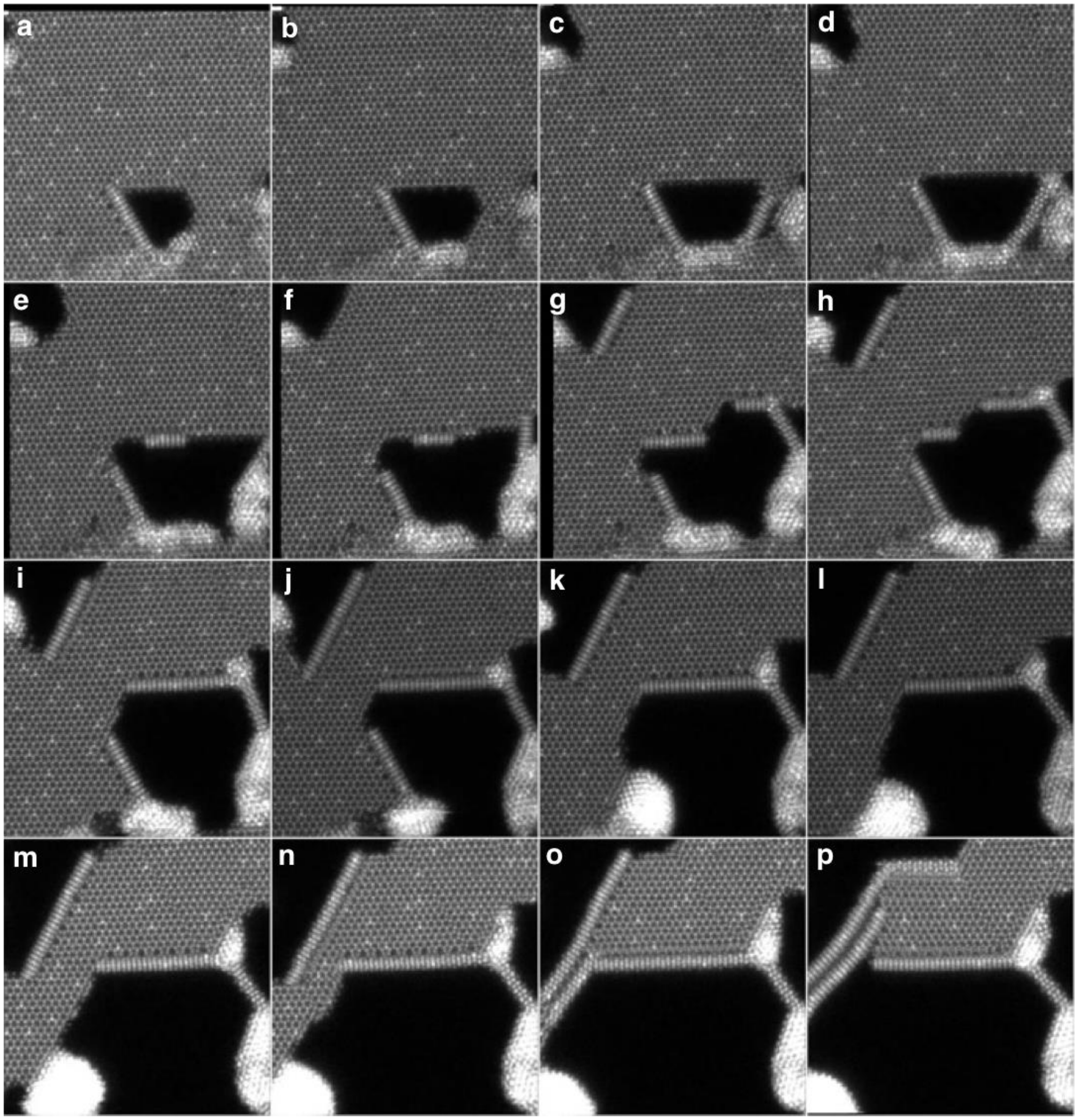

Supplementary Figure 18 | Shape evolution and edge reconstruction of etched pores at 500 °C. From **a**–**p**, the time interval between acquisition of each HAADF-STEM image is 35 s. The NW-terminated edges tend to move along the longitudinal direction, which protects the edges from lateral etching.

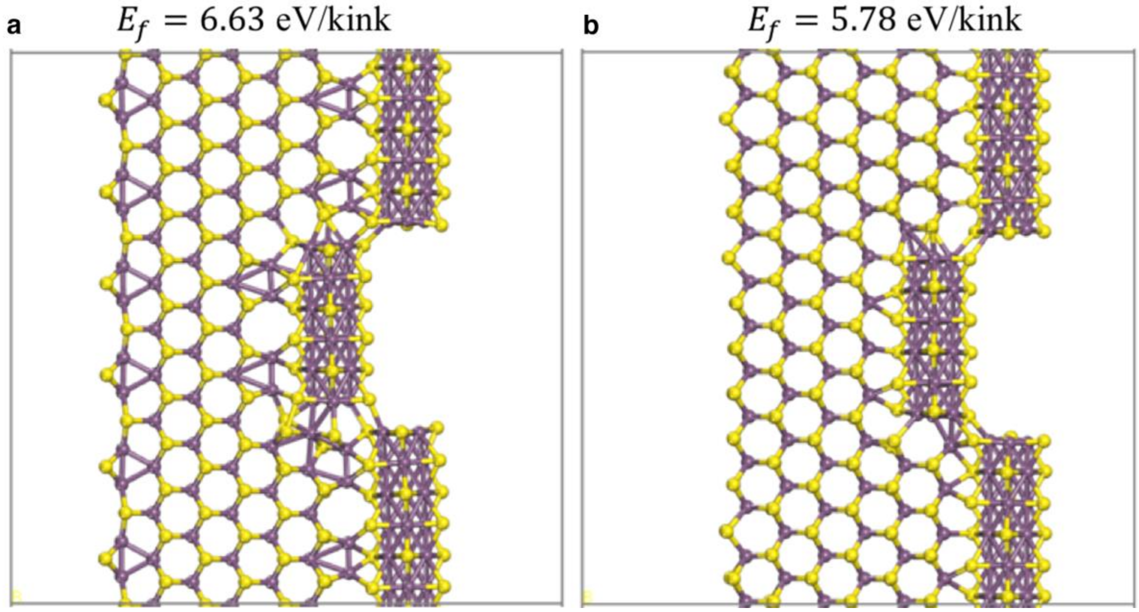

Supplementary Figure 19 | Formation energies of kinks in ZZSe-NW and ZZMo-NW edges. a-b, Relaxed structures of ZZSe-NW (**a**) and ZZMo-NW (**b**) edges with kinks. The formation energies to initiate a kink are annotated.

Supplementary Note 1

The formation energy of reference edges

Because the two edges of a zigzag $MoSe_2$ ribbon are different, one ZZSe and the other ZZMo, the formation energy of a ribbon always contains the edge energy of both ZZSe edge and ZZMo edge. Therefore, for example, when we need to compare the stability of ribbons with different ZZSe edges, we need to make the other edges (ZZMo) uniform, which is the reference edge. Then, the total formation energy of the ribbon minus the edge energy of the reference edge, is the formation energy of the edge we need.

To calculate the formation energy of reference edges, we construct triangular domains with different sizes, as shown in Supplementary Fig. 8. Considering the variation of the number of Se atoms at the edges and the characteristics of two elementary components, the stability of edges depends on the chemical potential of the components. So, the formation energy of a triangular domain shown in Supplementary Fig. 8 can be written as $E_f = E(\mathrm{Mo}_x\mathrm{Se}_y) - x\mu_{\mathrm{Mo}} - y\mu_{\mathrm{Se}}$, where $E(\mathrm{Mo}_x\mathrm{Se}_y)$ is the calculated energies of the domain. μ_{Mo} and μ_{Se} are the chemical potential of Mo and Se atoms, respectively. Assuming that $\mu_{\mathrm{Mo}} + 2\mu_{\mathrm{Se}} = \varepsilon = -19.936$ eV/$MoSe_2$, where ε is the energy per formula unit of $MoSe_2$ monolayer, the equation will change to:

$$E_f = E(\mathrm{Mo}_x\mathrm{Se}_y) + (2x - \mathrm{y})\mu_{\mathrm{Se}} - x\varepsilon \qquad (1)$$

Intuitively, the formation energy of a triangular domain, E_f, is composed of two parts. One is a constant associated with the formation of three vertices of the triangle (ε_v). The other one is three edges (γ) which is linearly proportional to the length of the boundary (l). Among the two terms, ε_v and γ are functions of μ_{Se} since both the vertices and edges have non-stoichiometric structures. Therefore, E_f can be rewritten as

$$E_f = 3\varepsilon_v + 3l\gamma \qquad (2)$$

Now the calculation of boundary formation energy γ is transformed into a problem of eliminating the effect of the vertices, which can be done by taking the energy difference between similar triangle domains with different side lengths. Supplementary Figure 8 shows optimized triangular domains with the ZZMo and ZZSe edges.

For a given μ_{Se}, E_f is calculated from Eq. (1). From the fitting parameters of Eq. (2), the linear fit of E_f, we can obtain the formation energy of the edges γ, and the formation energy of the three vertices of the triangular domain ε_v.

After we obtain the edge energy, γ, of the two reference edges, we can get the edge energy of only one edge of each calculated ribbon:

$$\gamma = E(\mathrm{Mo}_x\mathrm{Se}_y) + (2x - \mathrm{y})\mu_{\mathrm{Se}} - x\varepsilon - \gamma_{\mathrm{reference}} \quad (3)$$

Supplementary Fig. 9-15 show the structures and formation energies of all reasonable edges after DFT relaxation. We consider different reconstructions near the edge (grain boundary with rhombus rings, 5-8-5 ring, Mo-Mo chain, Mo-Mo dimer and Se-Se dimer etc. and chains with local 1T phase), different terminated Se atoms, and different NW rotations with and without removing Se atoms from NW.

As μ_{Mo} increases, the most stable edge structure changes from the original Se terminated edge, to edges with reconstruction (e.g., GB4 and 1T), to edges terminated with a NW.
