# Supplementary material for: In situ edge engineering in two-dimensional transition metal dichalcogenides
Source: Nat Commun. 2018 May 24;9:2051. doi: 10.1038/s41467-018-04435-x (PMC5967346; doi:10.1038/s41467-018-04435-x)
Supplement: Supplementary file 3 — Description of Additional Supplementary Files [file 41467_2018_4435_MOESM3_ESM.docx]

**Description of Additional Supplementary Files**

File Name: Supplementary Movie 1

Description: The complete movie of edge evolution in Fig. 1d.

File Name: Supplementary Movie 2

Description: The complete movie of edge evolution in Fig. 1e.

File Name: Supplementary Movie 3

Description: The complete movie of edge evolution in Fig. 1f.

File Name: Supplementary Movie 4

Description: The complete movie of edge evolution in Fig. 3a-d.

File Name: Supplementary Movie 5

Description: The complete movie of edge structure evolution during AIMD simulation.
